# The impact of personalised risk information compared to a positive/negative result on informed choice and intention to undergo colonoscopy following colorectal Cancer screening in Scotland (PERICCS) - a randomised controlled trial: study protocol

**DOI:** 10.1186/s12889-019-6734-0

**Published:** 2019-04-16

**Authors:** Robert J. C. Steele, Jayne Digby, Julie A. Chambers, Ronan E. O’Carroll

**Affiliations:** 1 Division of Cancer Research, University of Dundee, Ninewells Hospital, Dundee, DD1 9SY UK; 2 Psychology, University of Stirling, Stirling, FK9 4LA UK

**Keywords:** Colorectal cancer, Informed choice, Bowel screening, Colonoscopy, Personalised risk information

## Abstract

**Background:**

In Scotland a new, easier to complete bowel screening test, the Faecal Immunochemical Test (FIT), has been introduced. This test gives more accurate information about an individual’s risk of having colorectal cancer (CRC), based on their age and gender, and could lead to fewer missed cancers compared to the current screening test. However, there is no evidence of the effect on colonoscopy uptake of providing individuals with personalised risk information following a positive FIT test.

The objectives of the study are:

1. To develop novel methods of presenting personalised risk information in an easy-to-understand format using infographics with involvement of members of the public
2. To assess the impact of different presentations of risk information on informed choice and intention to take up an offer of colonoscopy after FIT
3. To assess participants’ responses to receiving personal risk information (knowledge, attitudes to screening/risk, emotional responses including anxiety).

**Methods:**

Adults (age range 50–74) registered on the Scottish Bowel Screening database will be invited by letter to take part. Consenting participants will be randomised to one of three groups to receive hypothetical information about their risk of cancer, based on age, gender and faecal haemoglobin concentration: 1) personalised risk information in numeric form (e.g. 1 in 100) with use of infographics, 2) personalised information described as ‘highest’, ‘moderate’ or ‘lowest’ risk with use of infographics, and 3) as a ‘positive’ test result, as is current practice. Groups will be compared on informed choice, intention to have a colonoscopy, and satisfaction with their decision. Follow-up semi-structured qualitative interviews will be conducted, by telephone, with a small number of consenting participants (*n* = 10 per group) to explore the acceptability/readability and any potential negative impact of the risk information, participants’ understanding of risk factors, attitudes to the different scenarios, and reasons for reported intentions.

**Discussion:**

Proving personalised risk information and allowing patient choice could lead to improved detection of CRC and increase patient satisfaction by facilitating informed choice over when/whether to undergo further invasive screening. However, we need to determine whether/how informed choice can be achieved and assess the potential impact on the colonoscopy service.

**Trial registration:**

The trial is registered on www.isrctn.com on 08/12/2017. Registration no: ISRCTN14254582.

## Background

In Scotland, the guaiac Faecal Occult Blood Test (gFOBT) has recently been replaced by a quantitative Faecal Immunochemical Test for haemoglobin (FIT) in the bowel cancer screening programme, after a successful demonstration pilot of “FIT as a First-Line Test”. FIT will initially be used with a faecal haemoglobin concentration of 80 μg Hb/g faeces in order to achieve approximately 2% positivity, to mimic the screening algorithm based on the previous two-tier reflex gFOBT/qualitative FIT strategy. However, FIT (along with age/gender) could provide a more personalised risk of harbouring advanced neoplasia (colorectal cancer (CRC) or high risk adenoma), and thus empower people to make an informed decision about whether or not to undergo colonoscopy, by balancing the risks of the procedure (e.g. bleeding/bowel wall perforation) against the risk of missing a cancer. This is particularly important in CRC screening. Currently, about half of all cancers in the screened population are diagnosed in the interval between screening rounds, indicating that, at the current threshold, the test is only about 50% sensitive for colorectal cancer. Reducing the threshold would result in fewer cancers being missed but would increase the chance of a negative colonoscopy and the impact on the colonoscopy service may be unsustainable. Thus, we aim to investigate the potential implications of providing the screened population with a more informed choice to help establish the impact on colonoscopy services.

This early phase study aims to assess patient responses to personalised risk information about their estimated risk of having colorectal cancer. Screened participants will be randomised to receive hypothetical scenarios of personalised risk information based on age, gender and haemoglobin concentration; or to treatment as usual, i.e. a positive/negative test result based on approximately 2% positivity (i.e. the proportion of screened individuals with a positive result). The best methods to present this information will be determined by this study. Study arms will be compared on level of informed choice achieved and intention to undergo colonoscopy and satisfaction with the decision made. Thus, we will assess the potential impact on colonoscopy services of introducing personalised risk information and whether informed choice can improve patient outcomes/satisfaction. The results will be used to inform a full-scale randomised controlled trial (RCT) to evaluate uptake of colonoscopy for Scottish Bowel Screening programme participants. The intervention could be low-cost to deliver at scale, and thus prove cost-effective for introduction within the national screening programme.

In order to inform a large population-based study, we must first assess the optimal method of delivering information for a truly informed choice. There is a dearth of current evidence. A recent Cochrane systematic review examined the effect of personalised risk communication for informed decision-making in uptake of medical screening compared to general information [1]. Overall, providing a numerical risk score or a categorised risk (e.g. ‘low’, ‘medium’, ‘high’) increased informed choice (odds ratio: 3.65 (95% C.I. 2.13 to 6.23), for random effects and screening uptake (odds ratio: 1.15 (95% C.I. 1.02 to 1.29)). However, the included studies covered a wide range of screening tests and thus the results were heterogeneous. Of the studies involving CRC, only one used a calculated numerical risk score, leading to greater knowledge but non-significant *lower* intention and uptake; it did not report changes in informed choice [2]. Three studies used a categorised risk score; these did not assess knowledge or informed choice, but indicated a small, significant increase in uptake of screening [3–5]. The authors concluded that the evidence that personalised risk communication increases screening uptake is weak. Further, although some included colonoscopy, all of the reviewed studies involving CRC screening related to first line screening only.

There is thus no existing evidence on the effects of personalised risk information on uptake of colonoscopy following first line screening for CRC. Our study would fill this knowledge gap. It will develop a novel personalised risk information intervention to enable national bowel screening programme participants to make an informed choice about having a colonoscopy following a FIT result with detected haemoglobin, by weighing their personalised risk versus the risks/disadvantages of colonoscopy. The proposed study has the potential to: 1) provide individuals with truly informed choice based on their own risk level, 2) reduce the number of ‘missed’ interval cancers by offering colonoscopy at lower thresholds than the current cut-off, 3) improve patient outcomes and satisfaction. We will determine the best methods for achieving informed choice and, if successful, they would have the potential to be extended to other screening/treatment scenarios, and thus have much wider impact. The study outcomes would give indicative effects to inform a larger RCT designed to provide evidence on any impact on colonoscopy services.

This research will deliver new knowledge about the impact of personalised risk information on delivering informed choice for colonoscopy and has the potential to benefit NHS managers and policy makers, cancer charities, members of the public, behavioural scientists and academics interested in health behaviour and informed choice.

Providing patients with informed choice is a core NHS tenet yet there is little evidence on its impact on CRC screening uptake. We believe this research could lead to improved detection of CRC, improve health outcomes and save lives. If results are promising, it would be feasible to present risk information in the format(s) developed in this research project within the scope of the Scottish bowel screening programme in the future. Thus it has the potential to increase patient satisfaction by helping them to make their own informed decision over when or whether to undergo invasive screening. Although there could be increased costs to the NHS due to increased colonoscopy uptake; earlier cancer detection could ultimately reduce NHS treatment costs and would contribute towards meeting the aim of the Detect Cancer Early Programme to improve 5-year survival.

In the longer-term, the intervention could be adapted for other settings where individualised information may be available including medical screening, treatment decisions and in promoting lifestyle changes to reduce future health risks, and so it has the potential for much wider impact. It is proposed that any follow-up study should include GP interactions, as patients receiving a positive FIT result may seek advice from their GP in making an informed decision about colonoscopy.

The aims of this study are:

1. To develop methods of presenting personalised risk information to Scottish Bowel Screening Programme participants, in an easy-to-understand format using infographics.
2. To assess the impact of different presentations of risk information on informed choice and intention to take up an offer of colonoscopy after FIT.
3. To assess participants’ responses to receiving personal risk information, including knowledge, attitudes to screening and risk, and emotional responses including anxiety.

## Methods

A simple 3-arm RCT design will be used for an intervention to present different levels of personalised risk and its effect on informed choice and intention to take up an offer of colonoscopy. The three groups are: 1) numerical personalised risk information, 2) categorical risk information, 3) positive screening test result. This is a single centre study covering the whole of Scotland and based at NHS Tayside, Ninewells Hospital & Medical School in Dundee.

Table 1 lists the objectives, hypothesis and variables, analysis techniques and anticipated conclusions of the study.

**Table 1:** Study Matrix

Objective	Hypothesis	Variables	Techniques of analysis	Anticipated Conclusions
1) Level of informed choice (derived from knowledge, attitudes, informed subscore of Decisional Conflict Scale). 2) Development of novel, personalised risk information materials. 3) Intention to take up an offer of colonoscopy.	Providing personalised risk information will provide individuals with truly informed choice based on their own risk level and improve satisfaction in screening participants.	• Age • Gender • SIMD • Study arm • Previous screening history • Measures of informed choice: ° Knowledge ° Attitude ° Informed subscore (Decisional Conflict Scale) • Behaviour (intention to uptake colonoscopy) • Anxiety	Comparison of knowledge, attitudes and intention to uptake colonoscopy between the study arms. Data on intention to take up colonoscopy from each scenario will be matched with the expected range of risk scores in the Scottish population. Comparison of themes from the qualitative analysis between those who intended and did not intend to take up the offer of colonoscopy. Any differences with respect to age/gender/ socioeconomic status and also whether or not previously participated in screening and/or taken up the offer of colonoscopy would also be explored.	Differences in intention to uptake colonoscopy between study arms will help to inform a full-scale RCT to evaluate uptake of colonoscopy in Scottish Bowel Screening Programme participants.

*SIMD* Scottish Index of Multiple Deprivation

Recruitment will be carried out by the Research Fellow (RF). Adults registered on the Scottish National Bowel Screening Programme database (age range 50–74 years) will be purposely selected to represent all age ranges, all deprivation categories and both genders as these factors have been shown to be associated with screening uptake. As it is typical in questionnaire-based research to get more responses from some groups (e.g. older people and higher socio-economic groups), the target recruitment will be weighted towards those groups who may be less likely to participate, based on response levels obtained from our large-scale questionnaire study on bowel screening [6], with the aim of achieving a balanced sample of participants.

Prospective participants would be approached, by letter, sent on behalf of the Principal Investigator (PI), Director of the Scottish Bowel Screening Programme, to ask if they would like to participate in a survey to assess their response to an offer of colonoscopy in relation to an estimated personalised risk of having cancer. A full patient information sheet and a response form will be included along with a pre-paid envelope for return. In line with published criteria for increasing recruitment [7], we would use techniques including personally addressed invitation letters, signed by the PI, stamped rather than franked return envelopes, and coloured ink. The participants would also be asked whether or not they would consent to taking part in a short follow-up interview by telephone. A response form will give the option to provide a telephone number if they are happy to being contacted in this way along with the postal address to receive the study risk materials with the questionnaire. Those not replying are assumed to have declined to participate. On receipt of the returned completed response form, it would be considered that the patient is in the study and would be randomised to receive the study materials. Return of the completed questionnaire will be considered as an implied consent.

Verbal consent will be confirmed at the start of the telephone interview, including consent for audio-recordings. The consent form will be read and audio recorded along with patient replies which will also be ticked on paper. Participants can still take part in the telephone interview, even if they do not wish to be recorded, in which case responses would be recorded on paper. A study ID will be assigned prior to transcription to ensure the interview is anonymised. The consent form will be stored separately to the anonymised study data.

A designated contact person who is external to the study team has also been nominated if prospective participants wish to discuss any concerns that invitees may have regarding the study before giving consent. The Patient Information Sheet will make clear that participants may withdraw from the study at any time without giving a reason and their data will be removed from the study.

Based on previous research [8], it is expected that 25% will respond and be randomised, we expect a further 20% attrition between consent and questionnaire return.

The risk information materials will initially be pilot tested in around 30 participants from the screening database. Therefore, 144 will be invited, with 36 expected to respond and 30 completing the questionnaire.

**Fig. 1:**
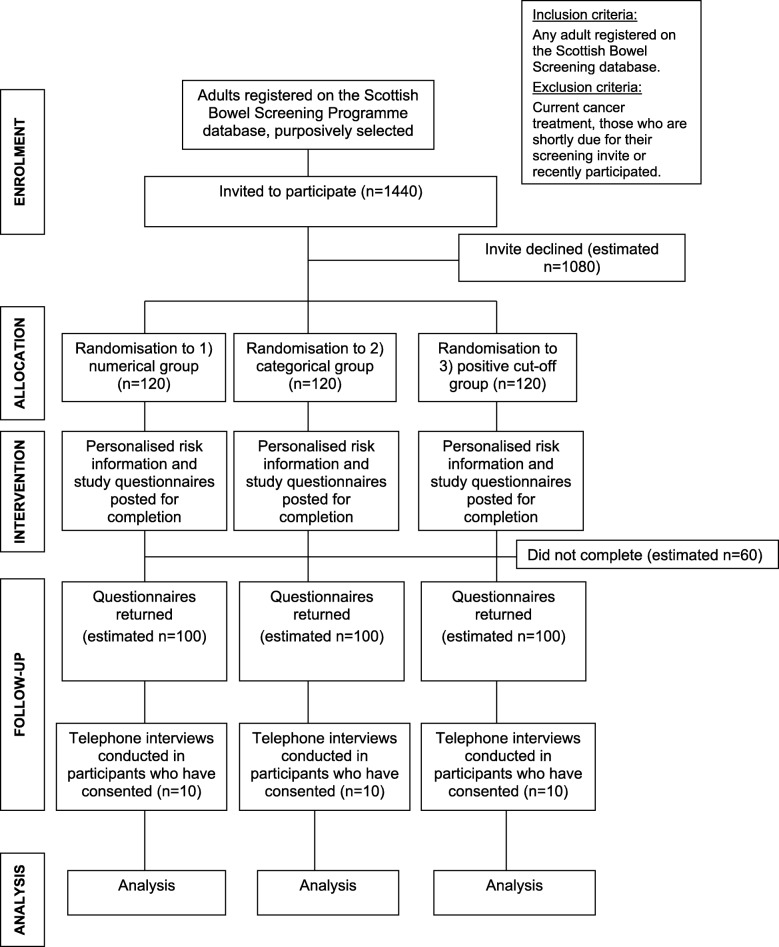
CONSORT diagram

Following the pilot testing in sample participants, a further 1440 will be invited to receive the finalised personalised risk information about their risk of having colorectal cancer and provide responses. As above, it is expected that 360 will respond and be randomised, with 300 then completing the study questionnaire. Figure 1 shows a flow diagram of the study design with expected numbers of participants. A small number of participants would be purposively sampled, from those consenting to be telephoned, from each treatment arm, estimated at 10 per arm, total = 30, to provide representation with regard to age, gender, socioeconomic status and responses to the scenarios.

Any adult registered on the Scottish Bowel Screening database with ability to consent (aged 50–74 years) will be included in the participant selection, but invitations will not be sent to those who have recently participated in screening, or will shortly be invited for screening (within six months of either event). This is to avoid any potential confusion between the study materials and the actual screening result.

For ethical reasons, the Study Participant Information Sheet will state that those currently being treated for cancer or other serious illness should ignore the invitation and accept our apologies for contacting them at what may be an inappropriate time. Inclusion on the screening database coincides with the first invitation at age 50, thus it is not possible to sample those not previously invited. Prospective participants would therefore include those who have previously been invited but not taken part in screening, those who have previously completed a gFOBt or FIT test, and also potentially a small number who have previously had a positive test and been offered colonoscopy.

Our aim is to develop novel, personalised risk information materials (for both risk of having CRC and risk of undergoing colonoscopy) which will be provided in a visual format (based on infographics [9]), aimed at being understandable across education levels, as guided by recommendations [10, 11]. It is planned to use absolute instead of relative risks, mortality instead of survival rates, and natural frequencies instead of conditional probabilities, as these are recommended as best practice [12]. However, this is open to change if the development process suggests otherwise.

The risk information materials for each of the study arms will be developed in months 1–12. We will recruit 2 Public and Patient Involvement (PPI) representatives to be core members of our project team and a further 4–6 PPI representatives to participate in co-development workshops along with the RF, the core project team and an infographics expert who has been engaged to design and produce the risk materials. The materials will then be pilot tested on sample participants before being refined and finalised.

The PPI participants will be from a healthy population group. They will be selected to be representative of the screening population with regard to socioeconomic status, age and gender. Those with a diagnosis of colorectal cancer will be excluded as it is important for the materials to be relevant to asymptomatic people who do not have a diagnosis of colorectal cancer. Medically qualified people will also be excluded. They will be sought via routes such as SHARE, the NHS Public Involvement Team and through other academic contacts with appropriate patient representative groups in place who might be interested in participating in this work.

The co-development workshops will be held throughout months 3–12 to develop, review and refine the project materials. We estimate that this would involve up to six three-hour meetings, which will be held at a venue convenient to the PPI group. Travel and subsistence costs will be met in full. As key members of the workshops, the PPI participants will contribute to the development process from a lay perspective, as health professionals and patients may form different interpretations of risk information. They will also provide feedback on the modified informed choice measure. We do not think it necessary to audio record the discussions; however, the RF will minute key points and circulate to the group following each meeting.

The risk materials will be pilot tested with a small sample (*n* = 30) of participants from the screening database, who would be posted the study invitation letter with reply slip, patient information sheet, risk information materials and informed choice questionnaire in advance of the main recruitment (month 7). An option to consent to telephone contact is available on the reply slip. Telephone interviews (months 8–9) will be conducted with consenting participants to assess ease of understanding and acceptability, and understanding of levels or risk based on the different scenarios. The impact on informed choice via the developed measure will also be assessed. The results will be fed into the finalisation of the materials, at a workshop involving the PPI members (month 10).

Presentation or risk will be assessed both as a ‘1 in x risk’ and natural frequencies in the development process, one or both of these may be used in the final materials. Participants will also be presented with the information on the risks of having a colonoscopy which is currently provided to those who are offered the test following a positive gFOBT. Group 3 is effectively a control group, which reflects current practice, to allow comparison of likely uptake of colonoscopy with the personalised risk information groups. Three verbal categories (lowest, moderate, highest risk) for simplicity of presentation of information are proposed. However, it is possible this could be modified during the development process, if the PPI involvement suggests otherwise. The development of the risk materials is a key part of the project, thus it is not possible to be too specific on detailed content or categorisations at this stage. The finalised risk materials will be used in the RCT across Scotland.

Patients who complete and return the response form indicating that they are willing to take part will be randomised to one of the study arms and then posted the materials and informed choice questionnaire with a stamped addressed envelope for return of the questionnaire. Study documents which will be sent to the participant are: Scenario letter(s) according to study arm, study questionnaire, “Having a colonoscopy” leaflet and “The Bowel Screening Test” leaflet. Return of the questionnaire will be considered as an implied consent.

Randomisation with minimisation on variables related to risk (i.e. age, SIMD, gender) will be carried out via MINIM software by an individual independent to the process of running the study, who will thus be blinded to the recruitment process.

The study will be co-ordinated by a Study Management Group, consisting of the PI, Co-investigators, Research Fellow and two PPI members.

Consenting participants will be randomised to one of 3 conditions:

1. Numerical (i.e. a personalised numerical-based risk assessment (e.g. 1 in x risk) related to hypothetical test result and age and gender)
2. Categorical (as treatment group 1) but this would be categorised as ‘Lowest’, ‘Moderate’ and ‘Highest’ risk
3. Positive cut-off (i.e. as current practice that is i.e. a positive test result, based on the current cut-off to give approximately 2% positivity).

Each condition would be presented with hypothetical scenarios relating to different levels of risk of CRC and asked to rate their intention of attending a colonoscopy if they received that result following an actual FIT. Three scenarios, relating to lowest, moderate and highest risk for colorectal cancer would be presented in groups 1 and 2; by definition group 3 has only one possible scenario i.e. a positive screening test result.

The lowest, moderate and highest risk categories and the disease frequencies used in the scenarios will be derived from data from a study which observed that risk of significant colorectal neoplasia rises with increasing faecal haemoglobin concentrations [13]. Although the actual faecal haemoglobin concentrations might represent different risks for individuals (e.g. different genders), the hypothetical scenarios would be presented as a specific level of risk (e.g. with this result you would have a 1 in x risk (or a moderate risk) of having CRC) and thus would be applicable to all participants. The aim of using both actual numerical risks and verbal categories is to assess their impact on the understanding and reactions of participants to these different forms of materials. The standard information describing the first-line screening process will be provided as an introduction to the different scenarios, as participants may have different previous experiences of first line screening.

A study questionnaire will be used to collect participant responses to information materials including intention, knowledge about the test (8 items: including risks of having cancer and undergoing a colonoscopy) and attitudes towards undergoing the test (4 items: including benefits, importance and pleasantness/ unpleasantness) and anxiety. Questionnaire data will be collected at a single time-point (months 19–21). Telephone interviews will be conducted in a small number of participants (10 from each study arm) to collect more detailed responses.

Data on screening history (i.e. previous participation/failure to participate/been offered a colonoscopy) will be collected at the time of sampling, as these strongly predict screening uptake [6]. We accept that recruitment via invitation letter may lead to a bias towards previous screening participants in our sample. However, in actual practice, the offer of informed choice following FIT would only be made to those who had completed the test with detected blood in their sample, so we believe this would be an acceptable, if not desirable, bias. However, as FIT has just been introduced in Scotland and the previous gFOBt is thought to be regarded as more difficult to complete [14], we would not want to exclude people who may complete a FIT in the future. Any differences with regard to previous screening history would be explored in our analysis (see below).

There is as yet no accepted measure of informed choice in cancer screening uptake [12]. Our *informed choice* measure will be adapted from Smith et al. [9]. They applied a multidimensional model of informed choice (developed and validated for antenatal screening for Down’s syndrome [15, 16], and combined the constructs of a) knowledge, b) attitudes and c) behaviour to assess the extent to which people made an informed choice about participating in screening using gFOBT. Items measuring knowledge about the test (8 items: including risks of having cancer and undergoing a colonoscopy) and attitudes towards undergoing the test (4 items: including benefits, importance and pleasantness/ unpleasantness) will be adapted to relate to colonoscopy rather than gFOBT. As this is therefore a new scale, the measure will be pilot tested with the PPI project participants, workshop participants and a small sample of participants from the bowel screening database (the same sample as in development of risk materials above) to assess acceptability and understanding. Full psychometric testing of the developed measure will be undertaken and reported.

We will use the informed subscore (items 1–3) from the Decisional Conflict Scale [17] to assess the extent to which participants feel informed about their decisions. These items are: “I know which options are available to me”, “I know the benefits of each option” and “I know the risks and side effects of each option”, scored on a 7-point scale from “strongly agree” to “strongly disagree”. As this is a hypothetical scenario study we are only able to use intention and not actual uptake as our outcome measure of behaviour. However, intention was very strongly related to actual uptake (with a mean intention score of 6.7 out of a maximum 7 for kit returners) in our previous large-scale study [6].

*Intention* to take up the offer of colonoscopy, as a proxy for behaviour, will be measured on a Likert-type scale from 1 (low intention) to 7 (strongly intend) in response to e.g. “If I received information that I had *x* level of risk of bowel cancer, I would choose to have a colonoscopy”. *Anxiety* will be measured by a previously-validated six-item version of the State Trait Anxiety Inventory [18]. An example item is the statement “I am worried”, with a choice of four responses on a scale from “not at all” to “very much”.

Ease of understanding and acceptability of the presentation of risk information will also be assessed in the questionnaire using Likert-type questions e.g. “I found the information presented easy to understand” scored on a 7-point scale from “strongly agree” to “strongly disagree”.

A small number of participants would be purposively sampled, from those agreed to be telephoned/consented for the interview, from each treatment arm (estimate *n* = 10 per arm, total *n* = 30) to provide representation with regard to age, gender, socioeconomic status and responses to the scenarios. The RF would conduct semi-structured interviews, by telephone, to explore the acceptability/readability and any potential negative impact of the risk information, participants’ understanding of risk factors and attitudes to the different scenarios presented, and reasons for their reported intentions. As only a small number of the 300 participants will be interviewed at follow-up, the questionnaire will include an open-ended final question encouraging additional comments. The follow-up telephone interviews will include questions relating to emotional responses to the material and understanding of what is meant by the verbal risk categories, as well as asking how participants perceive the risk of undergoing a colonoscopy versus that of having cancer in the given scenarios.

Telephone interviews are an effective means of gathering data in health research provided challenges are addressed [19]; and are more practical and cost-effective than face-to-face interviews as participants will be from the whole of Scotland. Interviews will be recorded (with permission) and fully transcribed.

All questionnaires and other records will be identified in a manner designed to maintain participant confidentiality. All records will be kept in a locked filing cabinet with limited access to study staff only. Archiving of study documents will be for five years after the end of study.

Data integrity of questionnaire data will be enforced by valid and range checks at the time of data entry. Clinical information will not be released without the written permission of the participant, except as necessary for monitoring and auditing by the Sponsor or its designee. All electronic information will be stored on secure network computers held on a University of Dundee network drive accessed by a password protected device. Data management will be conducted in compliance with Tayside Medical Science Centre (TASC) Standard Operating Procedures (SOP) on Data Management, TASC SOP53 Data Management Systems in Clinical Research.

The Data Management System (DMS) will be Excel, as approved by Sponsor. The DMS will be based on the protocol for the study and individual requirements of the investigators. Information will only be collected that is required to meet the aims of the trial and to ensure the eligibility and safety of the participant. The trial database will be compliant with TASC SOP53 Data Management Systems in Clinical Research. The anonymised data will be imported from Excel for analysis using SPSS (see below).

The database is managed in line with all applicable principles of medical confidentiality and UK law on data protection, namely, the Data Protection Act 1998, which brought UK law into line with the EU Data Protection Directive. The Data Controller will be the University of Dundee and the Data Custodian will be the PI. Database lock will be conducted in compliance with TASC SOP32 Locking Clinical Study Databases.

The PI, co-investigators and all institutions involved in the study will permit study related monitoring, audits, and Research Ethics Committee review. The PI agrees to allow the Sponsor or, representatives of the Sponsor, direct access to all study records and source documentation.

Knowledge, attitudes and intention to take up colonoscopy will be compared between the groups, using pre-determined categorisations [12], to assess whether providing personalised information on CRC risk leads to informed choice. In addition, between group differences in outcomes on the scales relating to knowledge, attitudes and behaviour will be analysed as continuous variables via Analysis of Variance (ANOVA) and individual items will also be examined, as recommended by Ghanouni et al. [20]. Covariates in the analyses are age, gender, deprivation category, and history of FOBT/FIT screening, as obtained from the screening database. Data on personal or family history of CRC are not available in the screening database and therefore cannot be examined.

Data on intention to take up colonoscopy from each scenario will be matched with the expected range of risk scores in the Scottish population (from the demonstration pilot of FIT as a First Line Test), to estimate total uptake, and hence the potential impact on colonoscopy services for numerical versus categorical presentation of personalised risk information, in comparison to the current positive/negative cut-off. This will provide preliminary evidence of the effects of implementing informed choice following introduction of FIT on current colonoscopy services.

Themes from the qualitative analysis will be compared between those who intended and did not intend to take up the offer of colonoscopy, which would help in understanding how the personalised risk information for both CRC and colonoscopy is understood and received and also the underlying reasons for the choices made. Any differences with respect to age/gender/socioeconomic status and also whether or not previously participated in screening and/or taken up the offer of colonoscopy will also be explored.

Thematic qualitative analysis will be used to analyse responses gathered from the telephone interviews as it permits rich description of participants’ perceptions, feelings and experiences [21]. Each interview will be analysed on a line-by-line basis for key themes/patterns. Recruitment will continue until saturation of themes is reached. The planned recruitment of *n* = 30 to the qualitative interviews is based on numbers required for saturation in previous research by the authors, but there is scope to increase this if saturation is not achieved.

An *n* = 300 (100 in each group) will have 83.7% power of detecting a 1 point increase in knowledge (intervention versus control), and a 2 point difference in attitudes (based on existing study means/SDs [9, 15]), using a one-way ANOVA. A pilot sample of 60–100 per group is recommended to provide an estimate of an event rate [22] (e.g. screening uptake); so a sample of 300 would provide an indicative effect size of colonoscopy uptake for a future full-scale study. We would send out 1440 invitations, from whom we conservatively expect to get around 360 replies (25%) (based on previous research [8]); we expect a further 20% attrition between consent and questionnaire return, giving a final *n* = 300. In the event that we do not hit our target of 360 replies to the first letter, a second wave of invitation letters would be sent out (the number of these would be based on actual response to and the deficit from the first invitation). We accept that the sample size may not be sufficient to assess all interactions between covariates. However this is a pilot study and a major aim is to assess effect sizes to determine accurate sample sizes for a larger study; we believe we have sufficient power to achieve this aim.

There is a risk of causing upset to patients with cancer or patients who may be disturbed by thinking about their cancer risk. It is not possible to exclude such patients from the initial contact. We have worded the Participant Information Sheet to express our apologies for contacting anyone in inappropriate circumstances. The patient invitation letter provides contact details of an individual who is completely independent of the research who can help in this case and advise to whom a complaint can be made to if required. It will also be stressed that risk scenarios are purely hypothetical. It should be noted that all individuals who will be contacted are enrolled in screening and the invite would not be dissimilar to the Scottish Bowel Screening invite.

If there is a problem with any participant it will be recorded in an Adverse Event log in compliance with TASC SOP11v9.

The PI will seek approval for any amendments to the Protocol or other study documents from the Sponsor, Research Ethics Committee (REC) and NHS R&D Office(s). Amendments to the protocol or other study docs will not be implemented without these approvals.

In the event that the PI needs to deviate from the protocol, the nature of and reasons for the deviation will be recorded in the Case Report Form, documented and submitted to the Sponsor. If this necessitates a subsequent protocol amendment, this will be submitted to the Sponsor for approval and then to the appropriate REC and lead NHS R&D Office for review and approval. In the event that a serious breach of Good Clinical Practice (GCP) or protocol is suspected, this will be reported to the Sponsor Governance Office immediately.

Annual reporting will be conducted in compliance with TASC SOP 15: Preparing and Submitting Progress and Safety Reports in CTIMPs and Non-CTIMPs, as a condition of sponsorship and as a condition of a favourable opinion from a REC. An HRA Annual Progress Report for NCTIMPs will be prepared and submitted by the CI to REC, and copied to the Sponsor, on the anniversary date of the REC favourable opinion.

Any safety reports additional to Serious Adverse Event reports, for example, reports of a Data Monitoring Committee, will be sent by the CI to REC, with a Safety Report Form, and to the Sponsor.

The end of study is defined as database lock once all data collection is complete. The Sponsor, CI and/or the SC have the right at any time to terminate the study for clinical or administrative reasons. The end of the study will be reported to the Sponsor and REC within 90 days, or 15 days if the study is terminated prematurely. The PI will ensure that any appropriate follow up is arranged for all participants. A summary report of the study will be provided to the Sponsor and REC within 1 year of the end of the study.

Ownership of the data arising from this study resides with the study team and their respective employers. On completion of the study, the study data will be analysed and tabulated, and a clinical study report will be prepared.

The clinical study report will be used for publication and presentation at scientific meetings. Investigators have the right to publish orally or in writing the results of the study.

Summaries of results will also be made available to Investigators for dissemination within their clinical areas (where appropriate and according to their discretion).

## Discussion

This early phase study will examine patient responses to personalised risk information about their risk of having CRC. The best methods to present personalised risk information based on age, gender and faecal haemoglobin concentration will be determined and compared to current practice i.e. a positive/negative test result. We will test whether this can lead to truly informed choice, the effect on intention to have a colonoscopy and thus potential impact on colonoscopy services, and whether informed choice can improve patient outcomes/satisfaction and potentially save lives.

Our study will be based on hypothetical scenarios and intention, rather than actual uptake of colonoscopy. Thus, we expect to use the results to inform a full-scale RCT which will evaluate actual uptake of colonoscopy for Scottish Bowel Screening programme participants. If successful, the intervention could be low-cost to deliver at scale, and thus prove cost-effective for introduction within the national screening programme.

## Abbreviations

ANOVA: Analysis Of Variance
CRC: Colorectal cancer
FIT: Faecal Immunochemical Test
GCP: Good Clinical Practice
gFOBT: Guaiac Faecal Occult Blood Test
PI: Principal Investigator
PPI: Public and Patient Involvement
RCT: Randomised Controlled Trial
REC: Research Ethics Committee
SIMD: Scottish index of multiple deprivation
SOP: Standard operating procedures
TASC: Tayside Medical Science Centre
